# Impact of Treatment Regimens on Antibody Response to the SARS-CoV-2 Coronavirus

**DOI:** 10.3389/fimmu.2021.580147

**Published:** 2021-04-15

**Authors:** Yufeng Shang, Tao Liu, Jingfeng Li, Natasha Mupeta Kaweme, Xinghuan Wang, Fuling Zhou

**Affiliations:** ^1^ Department of Hematology, Zhongnan Hospital of Wuhan University, Wuhan, China; ^2^ Department of Urology, Zhongnan Hospital of Wuhan University, Wuhan, China; ^3^ Center for Evidence-Based and Translational Medicine, Zhongnan Hospital of Wuhan University, Wuhan, China; ^4^ Department of Orthopedics, Zhongnan Hospital of Wuhan University, Wuhan, China

**Keywords:** SARS-CoV-2, COVID-19, IgG, cancer, chloroquine/hydroxychloroquine

## Abstract

The coronavirus disease 2019 (COVID-19) is widely spread and remains a global pandemic. Limited evidence on the systematic evaluation of the impact of treatment regimens on antibody responses exists. Our study aimed to analyze the role of antibody response on prognosis and determine factors influencing the IgG antibodies’ seroconversion. A total of 1,111 patients with mild to moderate COVID-19 symptoms admitted to Leishenshan Hospital in Wuhan were retrospectively analyzed. A serologic SARS-CoV-2 IgM/IgG antibody test was performed on all the patients 21 days after the onset of symptoms. Patient clinical characteristics were compared. In the study, 42 patients progressed to critical illness, with 6 mortalities reported while 1,069 patients reported mild to moderate disease. Advanced age (*P *= 0.028), gasping (*P *< 0.001), dyspnea (*P *= 0.024), and IgG negativity (*P* = 0.006) were associated with progression to critical illness. The mortality rate in critically ill patients with IgG antibody was 6.45% (95% CI 1.12–22.84%) and 36.36% (95% CI 12.36–68.38%) in patients with no IgG antibody (*P* = 0.003). Symptomatic patients were more likely to develop IgG antibody responses than asymptomatic patients. Using univariable analysis, fever (*P *< 0.001), gasping (*P* = 0.048), cancer (*P* < 0.001), cephalosporin (*P* = 0.015), and chloroquine/hydroxychloroquine (*P* = 0.021) were associated with IgG response. In the multivariable analysis, fever, cancer, cephalosporins, and chloroquine/hydroxychloroquine correlated independently with IgG response. We determined that the absence of SARS-CoV-2 antibody IgG in the convalescent stage had a specific predictive role in critical illness progression. Importantly, risk factors affecting seropositivity were identified, and the effect of antimalarial drugs on antibody response was determined.

## Introduction

COVID-19 continues to spread both nationwide and globally rapidly. Previously, no effective antiviral therapy or measures to control the epidemic were available before the vaccine was initiated. Chloroquine/hydroxychloroquine was presumed effective against SARS-CoV-2 as it previously showed inhibitory potential against most coronaviruses, including SARS-CoV-1 (1, 2). The results of preliminary trials of chloroquine repurposing in the treatment of COVID-19 in China have been encouraging (3). Chloroquine/hydroxychloroquine was approved for the treatment of COVID-19. The Food and Drugs Administration (FDA) granted hydroxychloroquine and chloroquine use as an emergency COVID-19 therapy on March 28, 2020 (4). Consequent results proved that chloroquine/hydroxychloroquine caused serious adverse effects (5, 6). Further, studies investigating the use of hydroxychloroquine and chloroquine were withdrawn due to the unavailability of complete datasets, client contracts, and complete ISO audit reports for analysis (7, 8). Recently, hydroxychloroquine’s impact on the antibody response to SARS-CoV-2 has raised the attention of clinicians (9), owing to the belief that hydroxychloroquine is harmful and does not influence the outcome of patients hospitalized with COVID-19 (10, 11). A serologic antibody detection to SARS-CoV-2 plays a crucial role in diagnosing COVID-19 as a complementary approach for viral nucleic acid assays (12–14). In a previous study, our analysis concluded that COVID-19 with cancer had a lower IgG prevalence than patients without cancer (15). There is currently limited evidence on the impact of chloroquine/hydroxychloroquine and various treatment regimens on antibody response.

In this study, we analyzed the impact of antibody response on patient clinical outcome and determined the factors influencing IgG antibody prevalence.

## Methods

The study analyzed 1,111 patients with mild or moderate COVID-19 at first admission to Leishenshan Hospital, Wuhan, China, between February 8, 2020, to March 29, 2020. A. COVID-19 was confirmed by RT-PCR test for SARS-CoV-2 on all the patients hospitalized (16), with a follow-up serologic SARS-CoV-2 IgM/IgG test 21 days post symptom onset. The severity of COVID-19 illness was defined according to the Chinese Management Guidelines for COVID-19 (version 7.0) (17). COVID-19 patients were stratified as follows: mild (i.e., mild clinical symptoms without features of pneumonia on imaging), moderate (i.e., clinical symptoms such as fever, cough, with features of pneumonia on imaging), severe (i.e., dyspnea, respiratory rate ≥30/min, blood oxygen saturation ≤93%, the partial pressure of arterial oxygen to fraction of inspired oxygen ratio <300, and/or lung infiltrates >50% within 24 to 48 h), and lastly, critically ill patients (i.e., respiratory failure, septic shock, and/or multiple organ dysfunction or failure). In this analysis, non-critical COVID-19 illness included mild, moderate, and severe classification conforming to the 7th Edition.

According to the Chinese Management Guidelines for COVID-19 (version 7.0) (17), treatment strategies included the application of antiviral drugs, rational use of antimicrobial therapy, antimalarial drugs, systemic corticosteroid therapy, immunotherapy, traditional Chinese medicine, to mention a few. Anti-SARS-CoV-2 viral drugs included Alpha Interferon, Ribavirin, Arbidol, Lopinavir/Ritonavir. In present studies, antimicrobial therapies include cephalosporins, quinolones, macrolides, carbapenems, antifungals, and other antibacterial drugs. The choice of therapeutic drug depends on the presentation of the disease. Chloroquine should be used in adults aged 18–65 years. For those who weigh more than 50 kg, 500 mg each time, two times a day, for 7 days; for those who weigh less than 50 kg, 500 mg each time on the first and second days, two times a day, and 500 mg each time on the third to 7th days, each time, once a day.

IgM/IgG test kits included recombinant SARS-CoV-2 antigens (spike protein and nucleocapsid protein) labeled with magnetic beads (tested on a fully automated chemiluminescence immunoassay analyzer) or colloidal gold (test card), anti-human IgM monoclonal antibody, and anti-human IgG monoclonal antibody. These test kits were reported to have high sensitivity and specificity (15, 18). Given that antibody concentration is a continuous variable among patients, the test kit has a fixed negative and positive criteria/cut-off. It directly indicated a “negative” or “positive” result when the sample was tested. According to the manufacturers, the sensitivity and specificity are ~90 and >99% for IgM, and ~98 and ~98% for IgG. COVID-19 IgM/IgG test date and results, date of symptom onset, treatment, and outcomes were obtained from electronic medical records. An expertly trained team of physicians further reviewed the data. For the assessing IgG test results, each patient’s final test result was used in the analyses. Our study was approved by the institutional ethics board at Zhongnan Hospital of Wuhan University (No. 2020074).

### Statistical Analysis

We used the Chi-square test or Fisher’s exact test for categorical variables and the Mann-Whitney U test for continuous variables to compare differences between the IgG negative and IgG positive groups and to determine which groups progressed to critical illness by IBM SPSS software, version 25.0. Factors affecting IgG antibody response were evaluated using logistic regression model in univariate and multivariate analysis. A forward/condition procedure was used to determine the final model by entering significant variables (*P* < 0.05) and removing non-significant variables (*P* > 0.10) one at a time. The maximum number of iterations was set to 20. Hosmer-Lemeshow test was used to check model goodness of fitting. The studentized residual was saved. Cumulative mortality events were estimated using the Kaplan–Meier method and log-rank test to assess the statistical significance of the differences. All reported *P* values were two-sided at a significance level of 0.05.

## Results

### General Information of Patients With COVID-19

**Figure 1:**
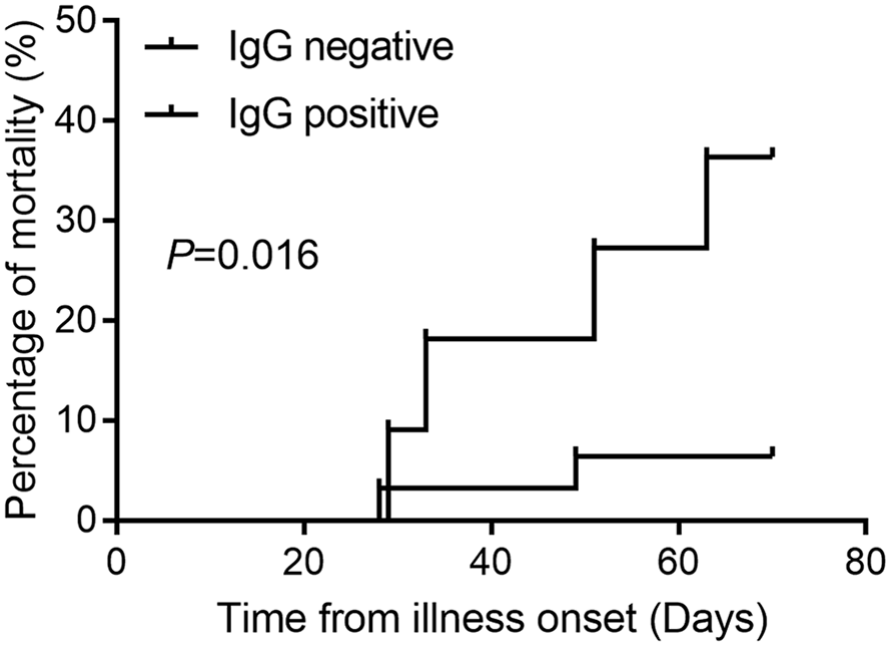
Cumulative mortality events in critical COVID-19 with and without IgG antibody response.

Among the 1,111 mild or moderate COVID-19 patients seen at first hospital admission, 42 patients progressed to critical illness, with six mortalities reported, while 1,069 patients did not progress to critical illness and survived. Only three patients were SARS-COV-2 RT-PCR positive and were classified as non-critical groups. The most common clinical symptom was fever (68.2%), cough (58.4%), followed by fatigue (25.6%), gasping (18.8%), and chest tightness (14.1%) (**Table 1**). Patients with gasping or dyspnea were more likely to progress to a critical illness (**Table A1**). In critical illness, the mortality rate in patients with IgG antibody was 6.45% (95% CI 1.12–22.84%), and 36.36% (95% CI 12.36–68.38%) in patients without IgG antibody (*P* = 0.016, **Figure 1**).

**Table 1:** Comparison of parameters between COVID-19 patients with and without IgG antibody.

Characteristics	All patients Number (%) or Median (IQR)	IgG Negative Number (%) or Median (IQR)	IgG Positive Number (%) or Median (IQR)	*P* value
**Age groups (years)**	57.0 (49.0–66.0)	61.0 (49.0–69.0)	57.0 (49.0–66.0)	0.106
≤65	801 (72.1)	83 (64.8)	718 (73.0)	0.052
>65	310 (27.9)	45 (35.2)	265 (27.0)	
**Sex**				0.804
Female	593 (53.4)	67 (52.3)	526 (53.5)	
Male	518 (46.6)	61 (47.7)	457 (46.5)	
**Days of hospitalization**	20.0 (14.0–26.0)	19.0 (11.0–23.0)	20.0 (14.0–27.0)	**0.005**
**Symptom**				**0.024**
Asymptomatic	32 (2.9)	8 (6.3)	24 (2.4)	
Symptomatic	1,076 (97.1)	120 (93.8)	956 (97.6)	
**Fever**	734 (68.2)	72 (60.0)	662 (69.2)	**0.040**
**Degree of fever**				**<0.001**
<37.3°C	343 (35.3)	48 (44.4)	294 (34.2)	
37.3–38.0°C	275 (28.4)	41 (38.0)	234 (27.2)	
38.01–39.0°C	274 (28.3)	18 (16.7)	256 (29.8)	
>39.0°C	77 (8.0)	1 (0.9)	76 (8.8)	
**Symptoms characteristics**				
Chills	20 (1.9)	3 (2.5)	17 (1.8)	0.481
Cough	628 (58.4)	72 (60.0)	556 (58.2)	0.700
Sore throat	35 (3.3)	4 (3.3)	31 (3.2)	0.999
Palpitations	15 (1.4)	0 (0.0)	15 (1.6)	0.397
Gasping	202 (18.8)	33 (27.5)	169 (17.7)	**0.009**
Chest pain	21 (2.0)	3 (2.5)	18 (1.9)	0.722
Chest tightness	152 (14.1)	23 (19.2)	129 (13.5)	0.093
Dyspnea	30 (2.8)	3 (2.5)	27 (2.8)	0.999
Dizziness	8 (0.7)	0 (0.0)	8 (0.8)	0.608
Headache	16 (1.5)	1 (0.8)	15 (1.6)	0.999
Fatigue	275 (25.6)	26 (21.7)	249 (26.0)	0.300
Diarrhea	32 (3.0)	3 (2.5)	29 (3.0)	0.999
Abdominal pain	6 (0.6)	2 (1.7)	4 (0.4)	0.137
Anorexia	27 (2.5)	3 (2.5)	24 (2.5)	0.999
Nausea or vomiting	6 (0.6)	1 (0.8)	5 (0.5)	0.509
Myalgia or arthralgia	30 (2.8)	4 (3.3)	26 (2.7)	0.766
**Cancer**				**<0.001**
Yes	19 (1.8)	8 (7.0)	11 (1.2)	
No	1,016 (98.2)	106 (93.0)	910 (98.8)	
**Treatment received**				
Anti-SARS-CoV-2 virus drugs	479 (43.1)	52 (40.6)	427 (43.4)	0.545
Antibiotics	331 (29.8)	50 (39.1)	281 (28.6)	**0.015**
Cephalosporin	114 (10.3)	22 (17.5)	92 (9.4)	**0.005**
Quinolone	255 (23.1)	33 (26.2)	222 (22.7)	0.378
Macrolide	9 (0.8)	3 (2.4)	6 (0.6)	0.073
Carbapenem	17 (1.5)	5 (4.0)	12 (1.2)	**0.036**
Antifungal	6 (0.5)	2 (1.6)	4 (0.4)	0.142
Other antibacterial drugs	25 (2.3)	7 (5.6)	18 (1.8)	**0.018**
Corticosteroids	74 (6.7)	6 (4.7)	68 (6.9)	0.341
Chloroquine/hydroxychloroquine	105 (9.5)	5 (3.9)	100 (10.2)	**0.023**
Vitamin C	174 (15.7)	25 (19.5)	149 (15.2)	0.200
Traditional Chinese Medicine	958 (86.2)	106 (82.8)	852 (86.7)	0.233

The significant P values are in bold.

### Comparison Between Patients Whether Progressed to Critical Illness or Not

For patients older than 65 years, 18 of 310 patients (5.81%) progressed to critical illness, while 24 of 801 patients (3.00%) aged 65 years and below progressed to critical illness (*P* = 0.028). Eleven of 128 patients (8.60%) progressed to critical illness in the IgG negative group, compared to that of 31 of 983 patients (3.15%) in the IgG positive group (*P* = 0.006). For treatment, patients progressing to critical illness had more prevalence of receiving antibiotics (*P* < 0.001) and corticosteroids (*P* < 0.001). One hundred (9.4%) of 1,069 patients that did not progress to critical illness received antimalarial drugs, and 5 (11.9%) of 42 patients who progressed to critical illness received antimalarial treatment (*P* = 0.587) (**Table A1**). A comparison of clinical characteristics between COVID-19 patients that progressed to critical illness and that did not progress to critical illness is presented in **Table A1**.

### Comparison Between COVID-19 Patients With and Without IgG Antibody

**Figure 2:**
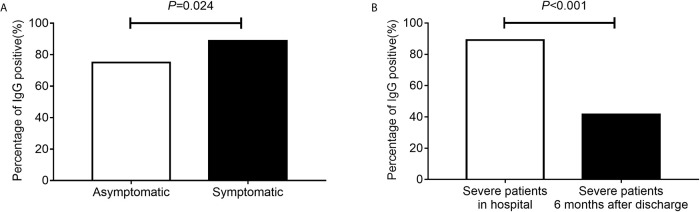
The percentage of IgG positive. The percentage of IgG positive in asymptomatic and symptomatic patients **(A)**. The percentage of IgG positive in severe patients during hospital and severe patients 6 months after discharge **(B)**.

The median time from symptom onset to antibody detection was 40 days (ranging from 21 to 87 days). IgG prevalence was 88.8% in symptomatic patients, and it was 75.0% in asymptomatic patients (**Figure 2A**, *P* = 0.024). A comparison of clinical parameters between COVID-19 patients with or without IgG antibody is illustrated in **Table 1**. The IgG positive rate was higher in patients with body temperature greater than 38°C, and patients with gasping had a lower IgG positive rate (**Table 1**). IgG prevalence was 57.9% (95% CI 34.0–78.9%) in cancer patients, and 89.6% (95% CI 87.5–91.4%) in non-cancer patients (*P* < 0.001). No statistical difference was observed in antibody response between different age groups (*P* = 0.052) or sex (*P* = 0.804). Of patients who received antibiotic therapy, 84.9% were IgG positive (*P* = 0.015), specifically, patients who achieve cephalosporin and carbapenem had a lower IgG positive rate (*P* = 0.005); and 95.2% of patients who received chloroquine/hydroxychloroquine therapy (*P* = 0.023) were IgG positive.

### The Clinical Characteristics of Patients With Cancers and COVID-19

There were 19 patients with cancers and COVID-19. The median age was 66.0 years (ranging from 42.0 to 79.0 years). Eight patients were male and 11 were female patients. There were three breast cancer, three lung cancer, two nasopharyngeal carcinoma, two renal cancer, two thyroid cancer, one liver cancer, one gastric cancer, one rectal cancer, one bladder cancer, one endometrial cancer, one laryngeal cancer, and one concurrent cancer of breast and lung cancer. The therapeutic strategies for tumors and the severity of COVID-19 were shown in **Table 2**. Nine patients progressed to severe illness and two patients progressed to critical illness. Eleven patients were IgG positive, and one case died.

**Table 2:** The basic information of patients with cancers and COVID-19.

Patient ID	Age (years)	Cancer type	Treatment	Admission severity	The worst severity	IgG	Survival status
P1	70–75	Nasopharyngeal carcinoma	Radiotherapy, Surgery	Mild	Severe	Positive	Survival
P2	76–80	Renal cancer	Surgery	Mild	Severe	Positive	Survival
P3	66–70	Bladder cancer	Surgery	Mild	Severe	Positive	Survival
P4	66–70	Liver cancer	Chemotherapy	Mild	Critical ill	Negative	Survival
P5	60–65	Gastric cancer	Surgery, Chemotherapy	Mild	Severe	Negative	Survival
P6	60–65	Breast cancer	Surgery, Chemotherapy	Mild	Moderate	Negative	Survival
P7	70–75	Laryngeal cancer	Radiotherapy, Chemotherapy	Mild	Severe	Positive	Survival
P8	66–70	Breast cancer, Lung cancer	Surgery, Chemotherapy	Moderate	Moderate	Negative	Survival
P9	70–75	Lung cancer	Chemotherapy	Moderate	Severe	Positive	Survival
P10	40–45	Nasopharyngeal carcinoma	Radiotherapy, Chemotherapy	Moderate	Critical ill	Negative	Death
P11	60–65	Thyroid cancer	Surgery	Moderate	Severe	Negative	Survival
P12	66–70	Lung cancer	Surgery, Radiotherapy, Chemotherapy	Moderate	Moderate	Positive	Survival
P13	66–70	Lung cancer	Radiotherapy,	Moderate	Severe	Positive	Survival
P14	56–60	Endometrial cancer	Surgery	Moderate	Moderate	Positive	Survival
P15	50–55	Thyroid cancer	Surgery	Moderate	Moderate	Positive	Survival
P16	46–50	Breast cancer	Surgery, Chemotherapy	Moderate	Moderate	Negative	Survival
P17	60–65	Renal cancer	Surgery	Moderate	Moderate	Positive	Survival
P18	66–70	Rectal cancer	Surgery	Moderate	Moderate	Positive	Survival
P19	50–55	Breast cancer	Surgery, Chemotherapy	Moderate	Severe	Negative	Survival

### Factors Associated With IgG Response

Factors associated with IgG response by univariable analysis and multivariable analysis were identified, adjusted by disease severity (**Table 3**). Using univariable analysis, fever (*P* < 0.001), gasping (*P* = 0.048), cancer (*P* < 0.001), chloroquine/hydroxychloroquine (*P* = 0.025), cephalosporin (*P* = 0.015), and other antibacterial drugs (*P* = 0.030) were associated with IgG response. In the multivariable analysis, fever, cancer, cephalosporin, and chloroquine/hydroxychloroquine remained independent correlation factors of IgG response. The significance of the Hosmer-Lemeshow test is 0.719, indicating that the model has a good fit. The distribution of residuals in relevant univariable was shown in **Figure A1**.

**Table 3:** Factors associated with IgG response by univariable analysis and multivariable analysis, adjusted by disease severity.

Characteristics	Univariable analysis		Multivariable analysis	
	OR (95% CI)	*P* value	OR (95%CI)	*P* value
Degree of fever		**<0.001**		**0.006**
<37.3°C	Reference	…	Reference	…
37.3–38.0°C	0.978 (0.630–1.518)	0.920	0.907 (0.558–1.473)	0.692
38.01–39.0°C	2.504 (1.431–4.381)	**0.001**	1.975 (1.104–3.533)	**0.022**
>39.0°C	16.046 (2.154–119.524)	**0.007**	12.376 (1.649–92.901)	**0.014**
Gasping	0.644 (0.416–0.997)	**0.048**	…	0.114
Cancer	0.169 (0.066–0.434)	**<0.001**	0.148 (0.048–0.456)	**0.001**
Chloroquine/Hydroxychloroquine	2.858 (1.138–7.178)	**0.025**	3.518 (1.213–10.201)	**0.021**
Cephalosporin	0.528 (0.315–0.885)	**0.015**	0.466 (0.260–0.836)	**0.010**
Carbapenem	…	0.149	…	…
Other antibacterial drugs	0.364 (0.146–0.907)	**0.030**	…	0.673

The significant P values are in bold; 95% CI, 95% Confidence Interval; OR, Odds Ratio.

### Follow-up of IgG Antibody for Patients 6 Months After Discharge

In our hospital, patients with severe illness were followed up 6 months after discharge, including 60 cases of present study, of which 55 cases were IgG positive and 5 cases were IgG negative. After 6 months, only 23 (41.6%) of these 55 IgG-positive cases continued to be IgG positive. Compared with the IgG detected during hospitalization, the difference in IgG positive rate was statistically significant (**Figure 2B**, *P* < 0.001). Besides, five cases of patients with IgG negative in hospital remained negative.

## Discussion

We tracked serological SARS-CoV-2 IgG antibody markers for 87 days, 21 days post symptom onset (convalescent stage) in 1,111 COVID-19 patients, and found that the specific antibody produced against SARS-CoV-2 was prevalent in 88.0% of patients, a result similar to other studies (19). Symptomatic patients were more likely to develop IgG antibody responses than asymptomatic patients, and this was consistent with the results of other studies (20, 21). These data suggested that asymptomatic individuals had a weaker immune response to SARS-CoV-2 infection. In present study, the prevalence of IgG was 41.6% 6 months after discharge. It was reasonable to speculate that the IgG might disappeared over time. The shedding of IgG might have implications for immunity strategy.

A lack of IgG antibody response in the convalescent stage was associated with a likelihood of progressing to critical illness and high mortality. Zhang et al. suggested that a higher titer of antibody was independently associated with a worsening clinical classification since 2-week after illness onset (22), while Yuen et al. and Gu et al. concluded that serum antibody did not correlate with clinical severity (23). Long et al. found that IgG titers in the severe group were higher than those in the non-severe group, but a significant difference only in the 2-week post-symptom onset group although a follow-up of >3-weeks was carried out (24). Disparate conclusions drawn from various studies may be due to the differences in the tracking period of antibody being analyzed. COVID-19 patients were seropositive to SARS-CoV-2 even at the early stage of illness (25), but an early rapid induction of antibody responses drove by high viral load contributed greatly to inflammatory responses, and was related to severe illness and poor prognosis (26). IgG antibodies usually take two weeks to develop and become high affinity protective antibody responses at late stage (13, 27). A significant neutralizing antibody response was observed in convalescent COVID-19 patients (21). Besides, almost 100% patients showed positive virus-specific IgG in early stage, and the antibody titers gradually increased during the first 3 weeks and then maintained (24). However, the IgG protective antibody may shed over time. Furthermore, patients infected tend to have a long SARS-CoV-2 carrying and infectious duration, and the persistent existence of SARS-CoV-2 could contribute to a worse disease and poor prognosis (28). This can reasonably be inferred that IgG only plays a protective role in the later stage, not in the early stage. In the case of the long-term existence of the virus, the shedding of antibodies in the later stage will not be conducive to the recovery of the disease even lead to the progression of disease.

The findings of Suthar et al. strongly indicated that a robust humoral immune response occurred early during severe or moderate COVID-19 infections (25). Early rapid induction of antibody response driven by high viral load leading to strong extrafollicular B cell responses was related to poor prognosis in the acute stage. The early detected antibodies that do not follow the sequence of IgM to IgG developmental stages significantly contribute to an inflammatory response by promoting monocyte and macrophage accumulation and massive cytokine storm and promote uptake of virion-antibody complex *via* Fc receptors (FcR), resulting in poor outcomes (26, 29). Wang et al. also observed that a positive correlation between NAb titers and SARS-CoV-2-specific IgG antibodies, and NAb levels were positively correlated with stem cell factor (SCF), TNF-related apoptosis-inducing ligand (TRAIL), and macrophage colony stimulating factor (M-CSF) levels during the acute phase (30). However, in the convalescent stage, the gradual development of viral antigen-specific B cells undergo somatic hypermutation and affinity maturation at the traditional germinal center, leading to high-affinity protective antibody responses (13). The humoral immune response is critical to the clearance of cytopathic viruses and is generally essential for preventing viral reinfection (31). Factually, factors related to adverse disease progression complicated disease outcomes. Excluding antibody, cytokine storm, aging, co-morbidities, to mention a few, also contributed to the clinical outcomes of COVID-19 patients (19, 32, 33).

There is limited literature reporting on the factors influencing antibody response. In a few similar studies, the prevalence of IgG in cancer patients was significantly lower, consistent with our conclusion (34). It remains unclear whether current or previous cancer treatments influence the immune response to the virus. Of the 19 cancer patients in our study, eight cases were IgG negative, and seven of eight patients underwent chemotherapy, except for one who only underwent surgery. Chemotherapy may play a role in immunosuppression. However, the hosts’ response to the SARS-CoV-2 may play a role in the COVID-19 course and representation due to differential immune cell profiles of cancer patients (35, 36). Notably, there is no research on the effect of therapeutic drugs, specifically chloroquine/hydroxychloroquine, on antibody response to COVID-19 (9). We concluded that the use of cephalosporin affected IgG antibody response (*P* = 0.010), and the prevalence of IgG in patients who received chloroquine/hydroxychloroquine was higher than in patients who did not receive chloroquine/hydroxychloroquine (*P* = 0.021).

Cephalosporins or some antibiotics were proved to affect the humoral and cellular immune response in animal (37–39). It was suggested that caution should be taken when administered the antibiotic during vaccination of animals (37). Chloroquine inhibits proteolysis, chemotaxis, phagocytosis, and antigen presentation in various professional antigen-presenting cells such as dendritic cells, B cells, and macrophages, leading to the inhibition of antigen-antibody reaction (40). The mechanism by which hydroxychloroquine increases the IgG antibody response is unclear. There is a hypothesized mechanism that chloroquine inhibited antigen degradation and improved the cross-presentation efficiency of dendritic cells, which suggested that chloroquine, followed by a booster dose of a soluble antigen immunization, can effectively enhance human T cell response (41–43). Both isotypes switch of antibody production, and the antibody affinity maturation requires T-cell help (44). The undetectable antibody response might because of inadequate T-cell helper response. Hydroxychloroquine shares the exact mechanism of action as chloroquine.

Chloroquine/hydroxychloroquine was restricted for use and was recommended to treat hospitalized COVID-19 positive patients in a clinical trial setting, with regard to shared and informed decision making together with patients, because of the undetermined therapeutic effect of chloroquine/hydroxychloroquine, such as cardiotoxicity, and additional adverse effects proven by prior clinical studies (5). Despite this limitation, chloroquine/hydroxychloroquine was considered the best therapeutic approach to impact the severity of SARS-CoV-2 infections in humans (45). Chloroquine/hydroxychloroquine promotes immunomodulatory effect *via* the production of cytokines and suppresses autophagy and lysosomal functionality in host cells (3), resulting in the production or maintenance of protective antibody IgG. However, the precise mechanism of action is not fully understood. Further study demonstrated that chloroquine/hydroxychloroquine had multiple effects on mammalian cells (46). When administered to virally infected hosts, it remains unclear what optimal dosage of chloroquine is needed to reduce unwanted tissue inflammation resulting from the anti-viral immune response to reduce overall disease severity or duration.

Our study has several limitations. We only included cases from a single hospital in Wuhan, and the results could not represent conclusions outside Wuhan and around the world. Analysis of a wider area is necessary. Moreover, viral load was not monitored in all the patients. Thus, we are undetermined whether the presence or absence of IgG antibody was influenced by the time of detection of viral RNA in throat swab samples in these patients. In this study, only three patients were SARS-CoV-2 RNA positive when the antibody was tested. It will be more consequential to analyze the relationship between viral load and antibody responses in future studies. Additional studies are needed to explore the dynamic changes between SARS-CoV-2 RNA and antibodies.

In conclusion, our study showed that not all COVID-19 patients had an IgG antibody response. Whether patients that had not developed antibodies may be re-infected by SARS-CoV-2 remains unknown. However, our study suggested that the absence of SARS-CoV-2 antibody IgG in the middle and late stages of COVID-19 had a specific predictive role in the progression of mild or moderate COVID-19 patients to critically ill states, which can help clinicians with early prediction and ascertain patient condition and guide treatment. Importantly, we determined the effect of antimalarial drugs on antibody response and identified risk factors affecting seropositivity to provide a reference basis for antibody response and future vaccinations.

## Data Availability Statement

The original contributions presented in the study are included in the article/**Supplementary Material**. Further inquiries can be directed to the corresponding authors.

## Ethics Statement

The studies involving human participants were reviewed and approved by Zhongnan Hospital of Wuhan University. The ethics committee waived the requirement of written informed consent for participation.

## Author Contributions

YS analyzed the data, designed the images and wrote the final manuscript, TL and JL collected the data, NK modified the language, and FZ and XW designed the project, provided professional guidance, and revised the final manuscript. All authors contributed to the article and approved the submitted version.

## Funding 

This work was supported by the Key Project for Anti-2019 novel Coronavirus Pneumonia from the Ministry of Science and Technology, China (grant number 2020YFC0845500).

## Conflict of Interest

The authors declare that the research was conducted in the absence of any commercial or financial relationships that could be construed as a potential conflict of interest.

## Supplementary Material

Figure A1 The distribution of residuals in relevant univariable. The degree of fever **(A)**, Cancer **(B)**, Chloroquine/hydroxychloroquine **(C)**, Cephalosporin **(D)**.
