# Supplementary figures and images for: Impact of Treatment Regimens on Antibody Response to the SARS-CoV-2 Coronavirus
Source: Front Immunol. 2021 Apr 15;12:580147. doi: 10.3389/fimmu.2021.580147 (PMC8082543; doi:10.3389/fimmu.2021.580147)

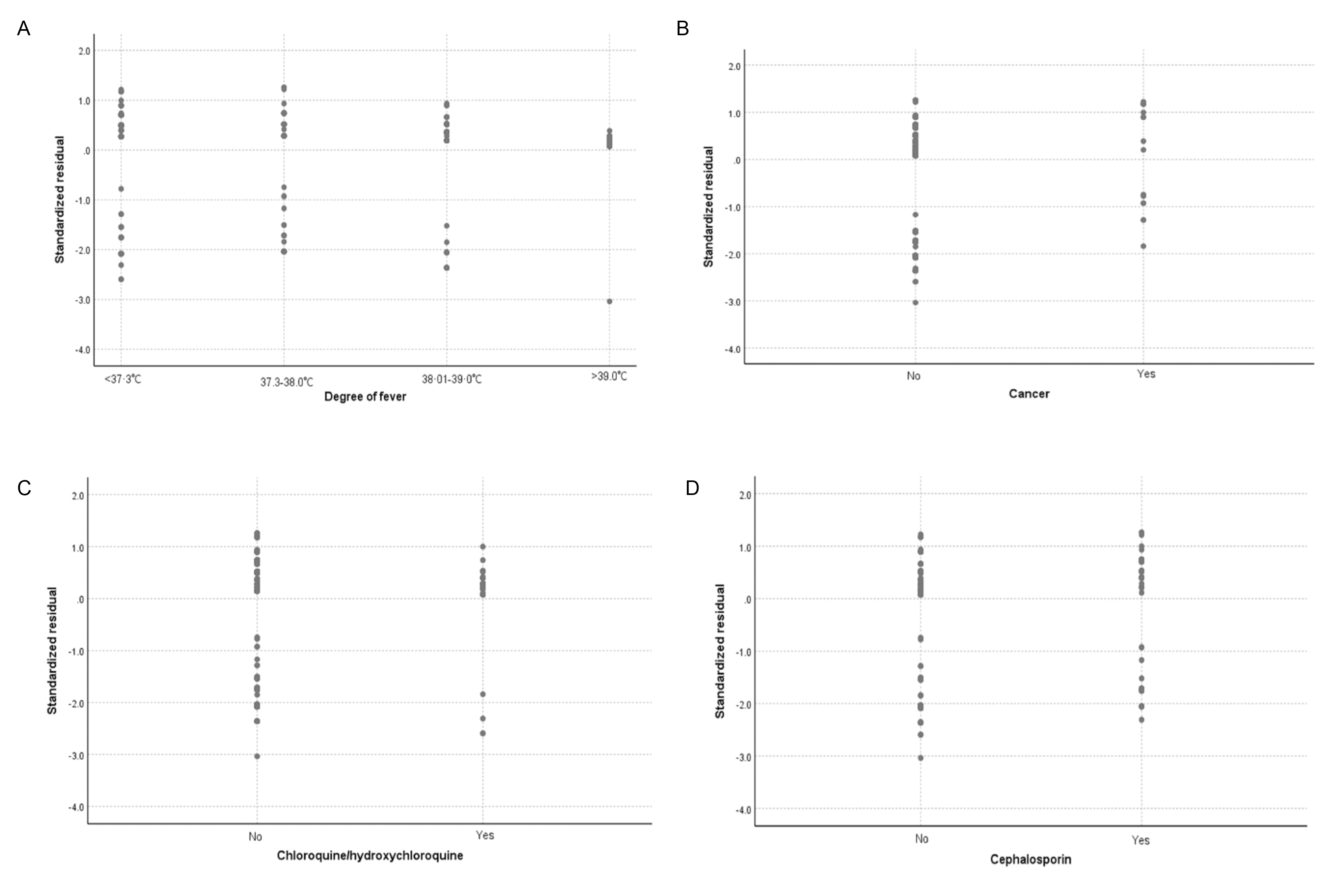

Supplement: Figure A1 — The distribution of residuals in relevant univariable. The degree of fever (A), Cancer (B), Chloroquine/hydroxychloroquine (C), Cephalosporin (D). [file Image_1.tif]
