# Supplementary material for: Impact of Treatment Regimens on Antibody Response to the SARS-CoV-2 Coronavirus
Source: Front Immunol. 2021 Apr 15;12:580147. doi: 10.3389/fimmu.2021.580147 (PMC8082543; doi:10.3389/fimmu.2021.580147)
Supplement: Supplementary file 2 [file Table_1.docx]

Table A1. Comparison of clinical characteristics associated with the incidence of progression to critical illness in COVID-19 patients.

| Characteristics | Progression to critical illness | | *P* value |
| --- | --- | --- | --- |
|  | No (n=1069)  Number (%) | Yes (n=42)  Number (%) |  |
| Age (years), Median (IQR) | 57.0 (48.0-66.0) | 62.0 (55.0-71.0) | **0.018** |
| Age groups (years) |  |  | **0.028** |
| ≤65 | 777 (72.7) | 24 (57.1) |  |
| >65 | 292 (27.3) | 18 (42.9) |  |
| **Sex** |  |  | 0.164 |
| Female | 575(53.8) | 18 (42.9) |  |
| Male | 494(46.2) | 24 (57.1) |  |
| **Fever** | 705(65.9) | 29(69.0) | 0.677 |
| **Degree of fever** |  |  | 0.012 |
| <37·3℃ | 364(37.7) | 13(35.1) |  |
| 37·3-38·0℃ | 268(27.7) | 7(18.9) |  |
| 38·01-39·0℃ | 265(27.4) | 9(24.3) |  |
| >39.0℃ | 69(7.1) | 8(21.6) |  |
| **Symptoms characteristics** |  |  |  |
| Chills | 19(1.8) | 1(2.4) | 0.540 |
| Cough | 606(56.7) | 22(52.4) | 0.581 |
| Sore throat | 34(3.2) | 1(2.4) | 0.999 |
| Palpitations | 15(1.4) | 0(0.0) | 0.999 |
| Gasping | 184(17.2) | 18(42.9) | **<0.001** |
| Chest pain | 21(2.0) | 0(0.0) | 0.999 |
| Chest tightness | 143(13.4) | 9(21.4) | 0.136 |
| Dyspnea | 26(2.4) | 4(9.5) | **0.024** |
| Dizziness | 8(0.7) | 0(0.0) | 0.999 |
| Headache | 16(1.5) | 0(0.0) | 0.999 |
| Fatigue | 269(25.2) | 6(14.3) | 0.109 |
| Diarrhoea | 32(3.0) | 0(0.0) | 0.629 |
| Abdominal pain | 5(0.5) | 1(2.4) | 0.207 |
| Anorexia | 24(2.2) | 3(7.1) | 0.078 |
| Nausea or vomiting | 6(0.6) | 0(0.0) | 0.999 |
| Myalgia or arthralgia | 30(2.8) | 0(0.0) | 0.625 |
| **IgG antibody** |  |  | **0.006** |
| Negative | 117 (10.9) | 11 (26.2) |  |
| Positive | 952 (89.1) | 31 (73.8) |  |
| **Cancer** |  |  | 0.178 |
| No | 976 (98.3) | 40 (95.2) |  |
| Yes | 17 (1.7) | 2 (4.8) |  |
| **Treatment received** |  |  |  |
| Anti-virus | 459 (42.9) | 20 (47.6) | 0.548 |
| Antibiotics | 304 (28.4) | 27(64.3) | **<0.001** |
| Corticosteroids | 61 (5.7) | 13 (31.0) | **<0.001** |
| Chloroquine/hydroxychloroquine | 100 (9.4) | 5 (11.9) | 0.587 |
| Vitamin C | 168 (15.7) | 6 (14.3) | 0.803 |
| Traditional Chinese Medicine | 923 (86.3) | 35 (83.3) | 0.579 |
